# Predictive value of postoperative prognostic nutritional index trajectory for mortality outcomes after off-pump coronary artery bypass surgery: a retrospective cohort study

**DOI:** 10.3389/fnut.2025.1530651

**Published:** 2025-05-07

**Authors:** Myung Il Bae, Jae-Kwang Shim, Hye Sun Lee, Soyoung Jeon, Young-Lan Kwak

**Affiliations:** ^1^Department of Anesthesiology and Pain Medicine, Yonsei University College of Medicine, Seoul, Republic of Korea; ^2^Anesthesia and Pain Research Institute, Yonsei University College of Medicine, Seoul, Republic of Korea; ^3^Biostatistics Collaboration Unit, Yonsei University College of Medicine, Seoul, Republic of Korea

**Keywords:** prognostic nutritional index (PNI), objective nutritional index, off-pump coronary artery bypass (OPCAB), coronary artery bypass graft (CABG), trajectory analysis, malnutrition, nutrition monitoring, mortality

## Abstract

**Background:**

The prognostic nutritional index (PNI) has been widely used as a nutritional metric in patients undergoing cardiac surgery because of its ability to incorporate both nutritional and inflammatory statuses. However, while preoperative PNI is well-known for its predictability of outcomes after coronary artery bypass grafting (CABG), the prognostic value of postoperative PNI has rarely been evaluated. This study investigated the changes in postoperative PNI values following off-pump coronary artery bypass (OPCAB) surgery using a trajectory analysis method and analyzed its influence on mortality outcomes.

**Methods:**

We retrospectively analyzed the data of 983 patients who underwent OPCAB surgery. PNI values from postoperative days 1, 2, 3, and 1 month was analyzed using the trajectory method, and patients were grouped based on the patterns of change in PNI values. The 1-year and overall mortality rates were compared between PNI trajectory groups. Additionally, multivariable logistic regression analysis was performed to identify independent risk factors for 1-year all-cause mortality, and multivariable Cox regression analysis was conducted for overall mortality.

**Results:**

The trajectory analysis categorized patients into two groups: the “PNI-improved group,” characterized by a sharp increase in PNI values after surgery, and the “PNI-fixed group,” which exhibited minimal changes in PNI values. The PNI-improved group had significantly lower 1-year mortality (1.1% vs. 9.5%, *p* < 0.001) and overall mortality (16.9% vs. 42.4%, *p* < 0.001) compared to the PNI-fixed group. Furthermore, the multivariable regression analysis indicated that the PNI trajectory pattern was an independent predictor of 1-year mortality (odds ratio: 7.931, 95% confidence interval [CI]: 3.117–20.180, *p* < 0.001) and overall mortality (hazard ratio: 2.120, 95% CI: 1.579–2.845, *p* < 0.001).

**Conclusions:**

Patients who exhibited a significant increase in PNI values during the month following OPCAB surgery experienced significantly lower 1-year and overall mortality rates than those with minimal changes in postoperative PNI values. The PNI recovery pattern was identified as an independent predictor of both 1-year and overall mortality after adjusting confounding factors. Recognizing the recovery patterns of postoperative PNI values after OPCAB surgery may be valuable for screening patients at high risk for mortality.

## 1 Introduction

Malnutrition, a common problem in patients undergoing coronary artery bypass grafting (CABG) (1), negatively affects patient outcomes, particularly through the detrimental and reciprocal influence of the inflammatory response (2, 3). The role of nutrition in CABG surgery has been widely studied; however, its impact in the context of off-pump coronary artery bypass (OPCAB) remains relatively underexplored, with conflicting results. One study reported that preoperative nutritional status is associated with unfavorable outcomes and 1-year mortality in OPCAB patients (4). In another study, preoperative nutritional status did not influence immediate postoperative outcomes but had a significant impact on long-term mortality after OPCAB (5).

To estimate the nutritional status, objective nutritional indices, comprising readily available biomarkers, have become popular owing to their convenience and simplicity, compared with subjective nutritional evaluations (6). Furthermore, their prognostic roles have been validated in various subsets of patients (7–9). Among existing objective nutritional indices, the prognostic nutritional index (PNI) became popular because it incorporates both nutritional and inflammatory statuses by adding the lymphocyte count to the serum albumin level in its calculation, representing immune capacity and protein storage (10). Notably, preoperative PNI values are associated with mortality and major adverse events in patients with coronary disease (11), including those undergoing CABG (12–14). However, most previous studies evaluating the prognostic value of PNI in surgical patients have primarily focused on preoperative status, despite the high prevalence of postoperative malnutrition in these individuals (13, 15). Surgical trauma and inflammatory reaction induce impaired protein synthesis, increased nutrient consumption, and immunosuppression (16–18), resulting in serious fluctuations and declines in PNI values (13, 19). Furthermore, as the PNI theoretically reflects recovery from surgical insult, its clinical significance may be enhanced when the impact of postoperative PNI on prognosis is examined at multiple time points rather than through a single assessment; however, comprehensive evidence supporting this notion is currently lacking.

Trajectory projection analysis is an unbiased statistical modeling technique used to identify and cluster subphenotypes with common characteristics within heterogeneous data (20, 21). Accordingly, trajectory analyses of changing patterns of biomarkers in previous studies have provided critical information that static values measured at a single time point could not reveal (22, 23). Thus, we aimed to investigate the changing pattern of postoperative PNI values up to 30 days after surgery using trajectory analysis and its impact on mortality outcomes in patients undergoing OPCAB surgery.

## 2 Methods

This single-center retrospective study was approved by the Institutional Review Board of Yonsei University Health System Gangnam Severance Hospital (Seoul, South Korea) (approval number: 3-2023-0184, approval date: 12 July 2023). Owing to its retrospective nature, the requirement for informed consent was waived. The study was conducted in accordance with the principles of the Declaration of Helsinki.

### 2.1 Study population

We retrospectively analyzed the data of patients aged ≥18 years who underwent OPCAB surgery at the Severance Hospital (Seoul, South Korea) between January 2010 and February 2021. Patients without PNI data from days 1 to 3 after surgery were excluded. To collect data at 1 month after surgery, patients without PNI data between 20 and 60 days after surgery were also excluded.

### 2.2 PNI calculation

PNI values were calculated according to the following formula based on a previous study (10): PNI = 10 × serum albumin level (g/dL) + 0.005 × total lymphocyte count (cells/mm^3^).

PNI values on postoperative days (POD) 1, 2, and 3 were calculated using the serum albumin level and peripheral blood lymphocyte count at each time point. The “1-month PNI” was defined as the PNI value closest to POD 30 among the PNI values between 20 and 60 days after surgery.

### 2.3 Data collection

We collected data by reviewing the hospital's electronic medical records. Data regarding age, sex, body mass index (BMI), preoperative medication, and preoperative comorbidities, such as hypertension, diabetes mellitus, cerebrovascular accident, chronic renal failure, chronic obstructive pulmonary disease (COPD), congestive heart failure, acute myocardial infarction (MI) within 1 month, and anemia, were collected. Acute MI was defined according to the fourth universal definition by the Joint Task Force (24). Additionally, we collected information on the European System for Cardiac Operative Risk Evaluation II (EuroSCORE II) scores, left ventricular ejection fraction, and preoperative laboratory results. These laboratory results included peripheral blood lymphocyte count, white blood cell count, platelet count, and serum levels of hemoglobin, albumin, glucose, creatinine, and creatine kinase-MB. We also gathered the following operative data: emergency status, number of grafts, operative time, intraoperative fluid input, intraoperative urine output, estimated bleeding volume, and perioperative transfusion requirements.

Regarding postoperative data, serum albumin concentration and peripheral blood lymphocyte counts from POD 1 to POD 3 and 1 month after surgery were collected. Serum albumin concentration was measured using either an Atellica CH930 (Siemens, Marburg, Germany) or Cobas c702 (Roche, Mannheim, Germany) device, while peripheral blood lymphocyte count was measured using either a Siemens Advia (Siemens, Marburg, Germany) or Sysmex XN (Sysmex, Kobe, Japan) system. Additionally, we collected information on the length of stay in the intensive care unit (ICU) and hospital, as well as on acute kidney injury (AKI), cerebrovascular accidents, cardiac reoperation, prolonged mechanical ventilation (>24 h), deep sternal wound infection, and both 1-year and overall mortality rates. AKI was defined according to the Kidney Disease Improving Global Outcomes guidelines (25), and cerebrovascular accidents were defined according to the American Heart Association and American Stroke Association (26). Information on surgery, death, and last follow-up dates was also collected. Information regarding the date of death was obtained from the Ministry of the Interior and Safety of the Republic of Korea. The data were provided after excluding personal information in accordance with the relevant laws and were used only for statistical analysis.

### 2.4 Study endpoints

The primary endpoint was 1-year all-cause mortality after OPCAB surgery. The secondary endpoint was overall all-cause mortality after OPCAB surgery.

### 2.5 Statistical analysis

Statistical analyses were performed using IBM SPSS Statistics version 23 (IBM Corp., Armonk, NY, USA), R software version 3.6.0 (The R Foundation for Statistical Computing), and MedCalc version 22.014 (MedCalc Software, Bvba, Ostend, Belgium). The Kolmogorov–Smirnov test was used to evaluate the normality of continuous variables. Since no continuous variable in this study demonstrated a normal distribution, they were expressed as medians (interquartile ranges [IQR]). Dichotomous variables were expressed as numbers (percentages). The Mann–Whitney *U* test was used to compare continuous variables, while the chi-square or Fisher's exact test was used to compare dichotomous variables.

Trajectory analysis was conducted using the longitudinal k-means method from the “latrend” package (20) in R version 3.6.0. This specialized statistical method analyzes patterns in longitudinal data and clusters similar patterns (20, 21). In this study, postoperative PNI values on POD1, POD2, POD3, and 1 month after surgery were analyzed using this trajectory method. Since the longitudinal k-means method requires that the last measurement of variables occur at the same time point, the date of the “1-month PNI” was set to 30 days after surgery, reflecting the average postoperative period for 1-month PNI values. The number of clusters was determined to ensure appropriate representativeness and assignment probabilities. The estimated assignment probabilities for the trajectory clusters are summarized in Supplementary Table S1. When classified into two clusters, each cluster had a sample size of over 300 patients, ensuring representativeness, and each cluster demonstrated an average assignment probability of over 90%. Therefore, trajectory analysis was conducted by classifying the data into two clusters.

Logistic regression analysis was performed to investigate the risk factors for 1-year mortality. Multivariable logistic regression analysis included variables with *p* < 0.05 from the univariable analysis. The variance inflation factor was calculated to assess multicollinearity. Since the PNI, chronic renal failure, and anemia were included in the regression model, peripheral blood lymphocyte counts, serum albumin level, creatinine level, hemoglobin level, and transfusion requirements were excluded to prevent multicollinearity. The EuroSCORE II was included in the multivariable model, while variables used in the EuroSCORE II calculation, such as COPD and left ventricular ejection fraction, were excluded from the model to avoid multicollinearity. “Post-PNI,” defined as the highest PNI value between postoperative days 1 and 3, was included in the multivariable model to represent immediate postoperative PNI values. Additionally, receiver operating characteristic (ROC) curve analysis of the multivariable regression model was performed. The areas under the ROC curve (AUROC) of the multivariable models were compared using Delong's method to determine whether the PNI trajectory pattern significantly improved the model's predictive power.

Cox regression analysis was performed to investigate the risk factors for overall all-cause mortality. The multivariable Cox regression model incorporated variables with *p* < 0.05 from the univariable analysis. The EuroSCORE II score was included in the multivariable model, while the factors used to calculate the EuroSCORE II score were omitted. Although age is included in the EuroSCORE II score calculation, it was added to the multivariable model due to its significant impact on overall mortality.

Kaplan–Meier curves representing 1-year and overall survival were plotted, and the log-rank test was performed to identify statistical differences between the groups. Statistical significance was set at *p* < 0.05.

## 3 Results

**Figure 1:**
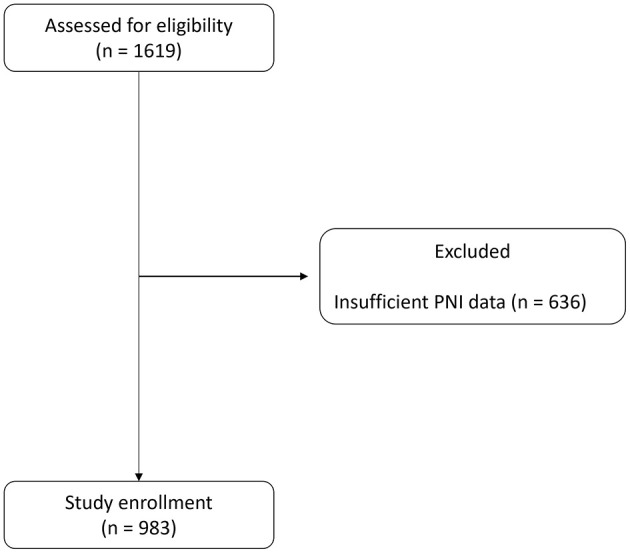
Flow diagram.

A total of 1,619 patients who underwent OPCAB surgery between January 2010 and February 2021 were screened for eligibility. Of these patients, 636 were excluded due to insufficient PNI data: 27 lacked immediate postoperative PNI data (POD 1–3), and 609 did not have 1-month PNI data (POD 20–60). Ultimately, 983 patients were included in the study (Figure 1). The median follow-up duration was 2,136 days (IQR 1,387–3,000).

**Figure 2:**
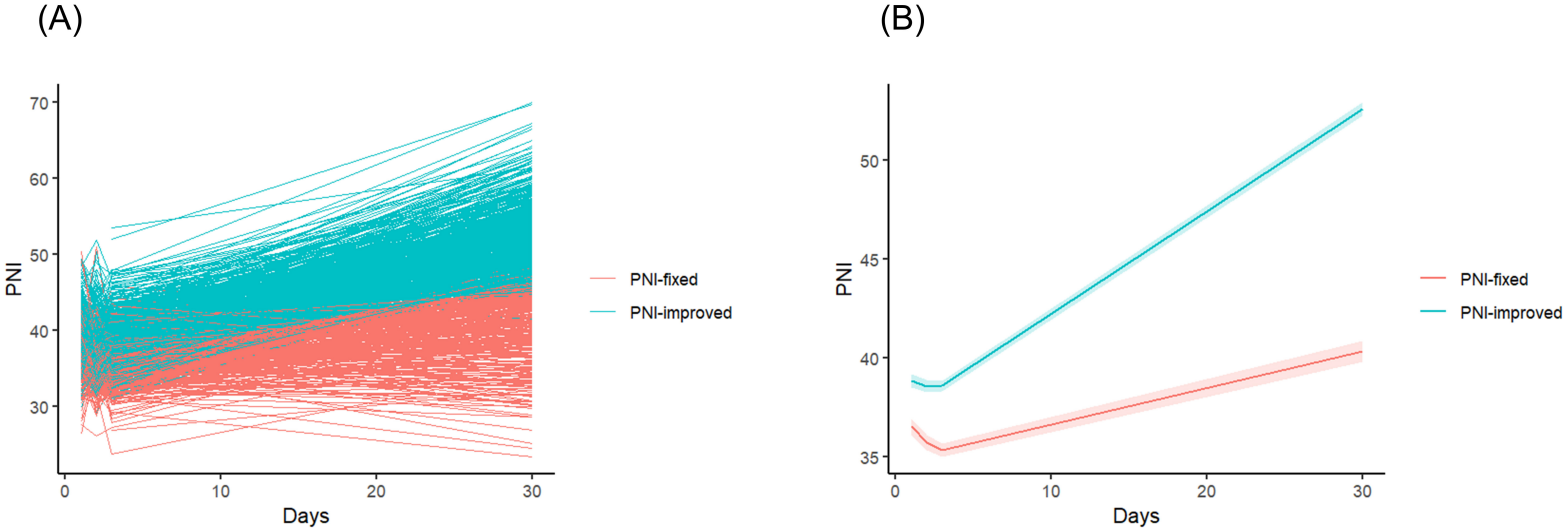
Trajectory analysis of PNI values after off-pump coronary artery bypass surgery. (A) Spaghetti plot showing the pattern of individual PNI value changes. The red line represents the cluster with minimal changes in PNI values after surgery (PNI-fixed group). The blue line represents the cluster with a steep increase in PNI values after surgery (PNI-improved group). (B) Mean profile plot according to trajectory clusters. The solid line represents the mean PNI values, and the shaded area represents the 95% confidence interval. PNI, prognostic nutritional index.

The trajectory analysis results for postoperative PNI values are summarized in Figure 2. One cluster exhibited a steep increase in PNI values during the first month after surgery (number of patients = 646, average probability of assignment = 94.43%) and was named as the “PNI-improved group.” The second cluster, referred to as the “PNI-fixed group,” showed minimal changes in PNI values during the same period (number of patients = 337, average probability of assignment = 91.83%).

Patients in the PNI-fixed group were older (68 [62–74] vs. 66 [59–72] years, *p* < 0.001) and had lower BMIs compared to those in the PNI-improved group. The prevalence of hypertension, chronic renal failure, cerebrovascular accidents, congestive heart failure, COPD, recent MI, and anemia was higher in the PNI-fixed group. Preoperative PNI was also lower in the PNI-fixed group (44.4 [39.1, 48.6] vs. 50.9 [47.1, 54.8], *p* < 0.001), as were hemoglobin levels, platelet counts, and left ventricular ejection fraction. EuroSCORE II scores were higher in the PNI-fixed group (1.67 [1.12, 2.80] vs. 1.08 [0.78, 1.69], *p* < 0.001), as was the frequency of perioperative transfusions (Table 1).

**Table 1:** Patient demographics, perioperative clinical data according to the PNI trajectory groups.

**Variables**	**Total (*N* = 983)**	**PNI-fixed (*n* = 337)**	**PNI-improved (*n* = 646)**	***p*-value**
Sex (female)	242 (24.6%)	86 (25.5%)	156 (24.1%)	0.636
Age (years)	67 (60, 73)	68 (62, 74)	66 (59, 72)	<0.001
Body mass index (kg/m^2^)	24.2 (22.1, 26.3)	23.4 (21.5, 25.6)	24.5 (22.5, 26.8)	<0.001
Emergency	24 (2.4%)	12 (3.6%)	12 (1.9%)	0.101
**Comorbidities**				
Hypertension	736 (74.9%)	274 (81.3%)	462 (71.5%)	0.001
Chronic renal failure	177 (18.0%)	117 (34.7%)	60 (9.3%)	<0.001
Cerebrovascular accident	155 (15.8%)	68 (20.2%)	87 (13.5%)	0.006
Diabetes mellitus	538 (54.7%)	219 (65.0%)	319 (49.4%)	<0.001
Congestive heart failure	127 (12.9%)	60 (17.8%)	67 (10.4%)	0.001
COPD	45 (4.6%)	22 (6.5%)	23 (3.6%)	0.035
Recent MI	304 (30.9%)	122 (36.2%)	182 (28.2%)	0.010
Anemia	497 (50.6%)	242 (71.8%)	255 (39.5%)	<0.001
**Medication**				
Beta blocker	566 (57.6%)	198 (58.8%)	368 (57.0%)	0.590
Calcium channel blocker	393 (40.0%)	130 (38.6%)	263 (40.7%)	0.516
RAS inhibitor	534 (54.3%)	204 (60.5%)	330 (51.1%)	0.005
Statin	784 (79.8%)	267 (79.2%)	517 (80.0%)	0.766
Diuretics	248 (25.2%)	108 (32.0%)	140 (21.7%)	<0.001
**Preoperative data**				
PNI	49.0 (44.0, 53.4)	44.4 (39.1, 48.6)	50.9 (47.1, 54.8)	<0.001
Albumin (g/dL)	4.0 (3.6, 4.3)	3.7 (3.3, 4.1)	4.1 (3.8, 4.4)	<0.001
Lymphocyte (/μl)	1,750 (1,360, 2,220)	1,410 (1,120, 1,770)	1,960 (1,560, 2,383)	<0.001
EuroSCORE II	1.24 (0.85, 2.10)	1.67 (1.12, 2.80)	1.08 (0.78, 1.69)	<0.001
White blood cells (/μl)	6,300 (4,920, 7,750)	6,100 (4,780, 7,315)	6,455 (4,990, 7,990)	0.042
Hemoglobin (g/dL)	12.5 (11.1, 14.0)	11.6 (10.1, 12.9)	13.1 (11.9, 14.3)	<0.001
Platelet (x10^3^/μl)	214 (178, 256)	203 (160, 254)	217 (184, 258)	0.001
Glucose (mg/dL)	123 (101, 166)	126 (102, 176)	121 (100, 162)	0.187
Creatinine (mg/dL)	0.92 (0.76, 1.16)	1.04 (0.84, 1.91)	0.88 (0.74, 1.05)	<0.001
CK-MB (μg/L)	1.8 (1.3, 2.6)	2.0 (1.4, 3.2)	1.7 (1.2, 2.4)	<0.001
Ejection fraction (%)	56 (44, 66)	49 (39, 63)	59 (47, 67)	<0.001
**Operation data**				
Graft number	3 (3, 4)	3 (3, 4)	3 (3, 4)	0.421
Operation time (min)	230 (207, 254)	230 (209, 253)	230 (206, 255)	0.569
Fluid Input/Output (ml)^*^	1,760 (1,340, 2,250)	1,750 (1,300, 2,210)	1,790 (1,350, 2,270)	0.294
Bleeding (ml)	600 (440, 780)	620 (460, 800)	600 (428, 773)	0.157
Perioperative Transfusion	520 (52.9%)	225 (66.8%)	295 (45.7%)	<0.001

Values are median (interquartile range) or number (%). ^*^Fluid Input/Output was defined as intraoperative fluid input minus urine output. PNI, prognostic nutritional index; COPD, chronic obstructive pulmonary disease; MI, myocardial infarction; RAS inhibitor, renin-angiotensin system inhibitor; CK-MB, Creatine Kinase-MB.

The incidence of 1-year all-cause mortality (9.5% vs. 1.1%, *p* < 0.001) and overall mortality (42.4% vs. 16.9%, *p* < 0.001) was significantly higher in the PNI-fixed group than in the PNI-improved group. The PNI-fixed group also had significantly lower PNI values on POD1, POD2, POD3, and 1 month after surgery compared to the PNI-improved group. Additionally, the lengths of stay in the ICU and hospital were significantly longer, while the incidence of AKI, prolonged ventilation, and sternal infection was significantly higher in the PNI-fixed group than in the PNI-improved group (Table 2).

**Table 2:** Postoperative data according to the PNI trajectory groups.

**Variables**	**Total (*N* = 983)**	**PNI-fixed (*n* = 337)**	**PNI-improved (*n* = 646)**	***p*-value**
POD1-PNI	37.7 (35.5, 40.1)	36.5 (34.5, 38.5)	38.5 (36.3, 41.1)	<0.001
POD1-Albumin (g/dL)	3.3 (3.1, 3.5)	3.2 (3.0, 3.4)	3.3 (3.1, 3.5)	<0.001
POD1-Lymphocyte (/μl)	970 (750, 1,260)	815 (640, 1,123)	1,070 (840, 1,340)	<0.001
POD2-PNI	37.4 (35.0, 39.5)	35.7 (33.7, 37.7)	38.3 (36.4, 40.5)	<0.001
POD2-Albumin (g/dL)	3.1 (3.0, 3.3)	3.1 (2.9, 3.2)	3.2 (3.0, 3.3)	<0.001
POD2-Lymphocyte (/μl)	1,170 (885, 1,515)	960 (733, 1,200)	1,280 (1,035, 1,650)	<0.001
POD3-PNI	37.2 (35.0, 39.8)	35.4 (33.3, 37.1)	38.3 (36.1, 40.9)	<0.001
POD3-Albumin (g/dL)	3.1 (2.9, 3.3)	3.0 (2.9, 3.2)	3.1 (3.0, 3.3)	<0.001
POD3-Lymphocyte (/μl)	1,230 (940, 1,550)	950 (758, 1,263)	1,380 (1,100, 1,710)	<0.001
1month-PNI	49.6 (44.0, 53.5)	41.4 (37.1, 44.3)	52.2 (49.6, 55.2)	<0.001
1month-Albumin (g/dL)	4.0 (3.6, 4.3)	3.4 (3.0, 3.7)	4.2 (4.0, 4.4)	<0.001
1month-Lymphocyte (/μl)	1,780 (1,380, 2,270)	1,330 (1,060, 1,645)	2,040 (1,678, 2,410)	<0.001
ICU length of stay (days)	3 (3, 4)	3 (3, 5)	3 (2, 3)	<0.001
Hospital length of stay (days)	10 (8, 13)	12 (9, 20)	9 (8, 11)	<0.001
Acute kidney injury	267 (27.2%)	141 (41.8%)	126 (19.5%)	<0.001
Cerebrovascular accident	16 (1.6%)	9 (2.7%)	7 (1.1%)	0.062
Cardiac reoperation	19 (1.9%)	10 (3.0%)	9 (1.4%)	0.089
Prolonged ventilation	104 (10.6%)	59 (17.5%)	45 (7.0%)	<0.001
Sternum infection	73 (7.4%)	47 (13.9%)	26 (4.0%)	<0.001
One-year mortality	39 (4.0%)	32 (9.5%)	7 (1.1%)	<0.001
Overall mortality	252 (25.6%)	143 (42.4%)	109 (16.9%)	<0.001

Values are median (interquartile range) or number (%). POD, postoperative day; PNI, prognostic nutritional index, ICU, intensive care unit.

Table 3 shows the results of the multivariable logistic regression analysis for predicting 1-year mortality. The PNI trajectory pattern (odds ratio [OR]: 7.931, 95% confidence interval [CI]: 3.117–20.180, *p* < 0.001) and preoperative anemia (OR: 2.964, 95% CI: 1.126–7.797, *p* = 0.028) were identified as independent predictors of 1-year all-cause mortality following OPCAB surgery. The results of the univariable analysis are presented in Supplementary Table S2.

**Table 3:** Multivariable logistic regression analysis of chosen variables for predicting one-year mortality.

**Variable**	**Adjusted odds ratio (95% CI)**	***p*-value**
PNI trajectory pattern (PNI-Fixed)^*^	7.931 (3.117, 20.180)	<0.001
Pre-PNI	0.993 (0.935, 1.056)	0.832
Post-PNI	1.075 (0.966, 1.196)	0.186
EuroSCORE II	0.963 (0.819, 1.132)	0.645
Body mass index (kg/m^2^)	0.899 (0.804, 1.005)	0.061
Emergency	3.093 (0.785, 12.189)	0.107
Anemia	2.964 (1.126, 7.797)	0.028

^*^Odds ratio of the PNI-Fixed pattern compared to the PNI-Improved pattern. PNI, prognostic nutritional index.

**Figure 3:**
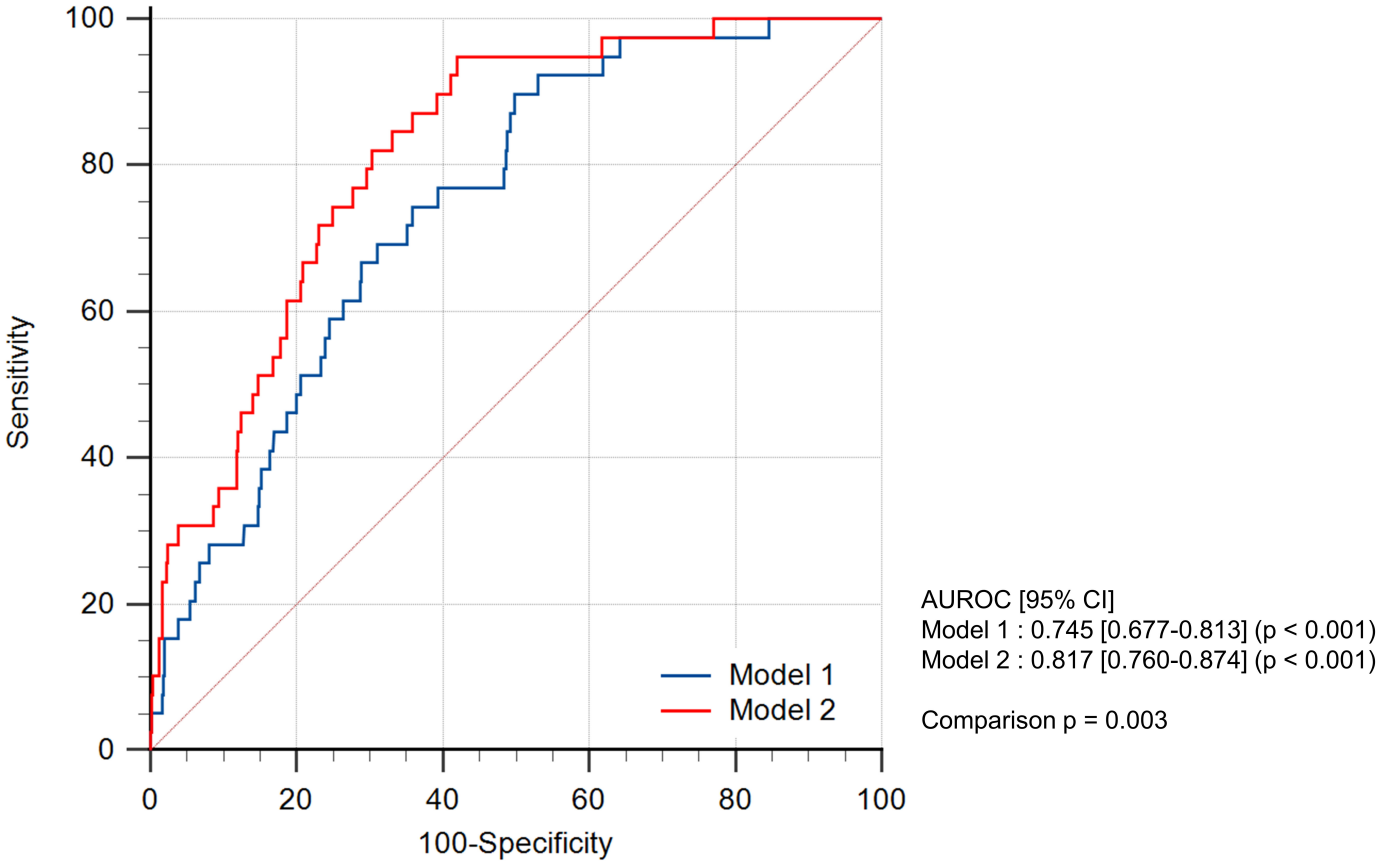
ROC curves for predicting 1-year mortality, derived from multivariable logistic regression models. This illustration shows the ROC curves derived from the multivariable logistic regression models predicting 1-year mortality. The blue line represents the curves of Model-1, which includes the pre-PNI, post-PNI, EuroSCORE II, BMI, emergency surgery, and anemia data. The red line represents the curves of Model-2, which includes the PNI trajectory pattern and the variables from Model 1. The AUROC of Model-2 was significantly higher than that of Model-1 (*p* = 0.003). PNI, prognostic nutritional index; ROC, receiver operating characteristic; AUROC, area under the receiver operating characteristic curve.

Figure 3 shows the ROC curves of the multivariable logistic regression models for predicting 1-year mortality. Model-1 comprises a multivariable model consisting of pre-PNI, post-PNI, EuroSCORE II, BMI, emergency surgery, and anemia, which excluded the PNI trajectory pattern from the model. Model-2 combined the variables from Model-1 with the PNI trajectory pattern. By adding the PNI trajectory pattern, the AUROC of Model-2 was significantly greater than that of Model-1 (0.817 [0.760–0.874] vs. 0.745 [0.677–0.813], *p* = 0.003).

Table 4 shows the results of the multivariable Cox regression analysis for predicting overall mortality. The PNI trajectory pattern (hazard ratio [HR]: 2.120, 95% CI: 1.579–2.845, *p* < 0.001), preoperative PNI (HR: 0.972, 95% CI: 0.950–0.996, *p* = 0.020), age (HR: 1.040, 95% CI: 1.024–1.057, *p* < 0.001), and preoperative anemia (HR: 1.558, 95% CI: 1.153–2.106, *p* = 0.004) were independent predictors of overall all-cause mortality. The results of the univariable analysis are presented in Supplementary Table S3.

**Table 4:** Multivariable Cox regression analysis of chosen variables for predicting overall mortality.

**Variable**	**Adjusted hazard ratio (95% CI)**	***p*-value**
PNI trajectory pattern (PNI-Fixed)^*^	2.120 (1.579, 2.845)	<0.001
Pre-PNI	0.972 (0.950, 0.996)	0.020
Post-PNI	1.012 (0.969, 1.057)	0.578
EuroSCORE II	1.055 (0.998, 1.115)	0.061
Age (years)	1.040 (1.024, 1.057)	<0.001
Body mass index (kg/m^2^)	0.985 (0.946, 1.027)	0.478
Anemia	1.558 (1.153, 2.106)	0.004
Graft number	0.939 (0.814, 1.084)	0.393

^*^Hazard ratio of the PNI-Fixed pattern compared to the PNI-Improved pattern. PNI, prognostic nutritional index.

**Figure 4:**
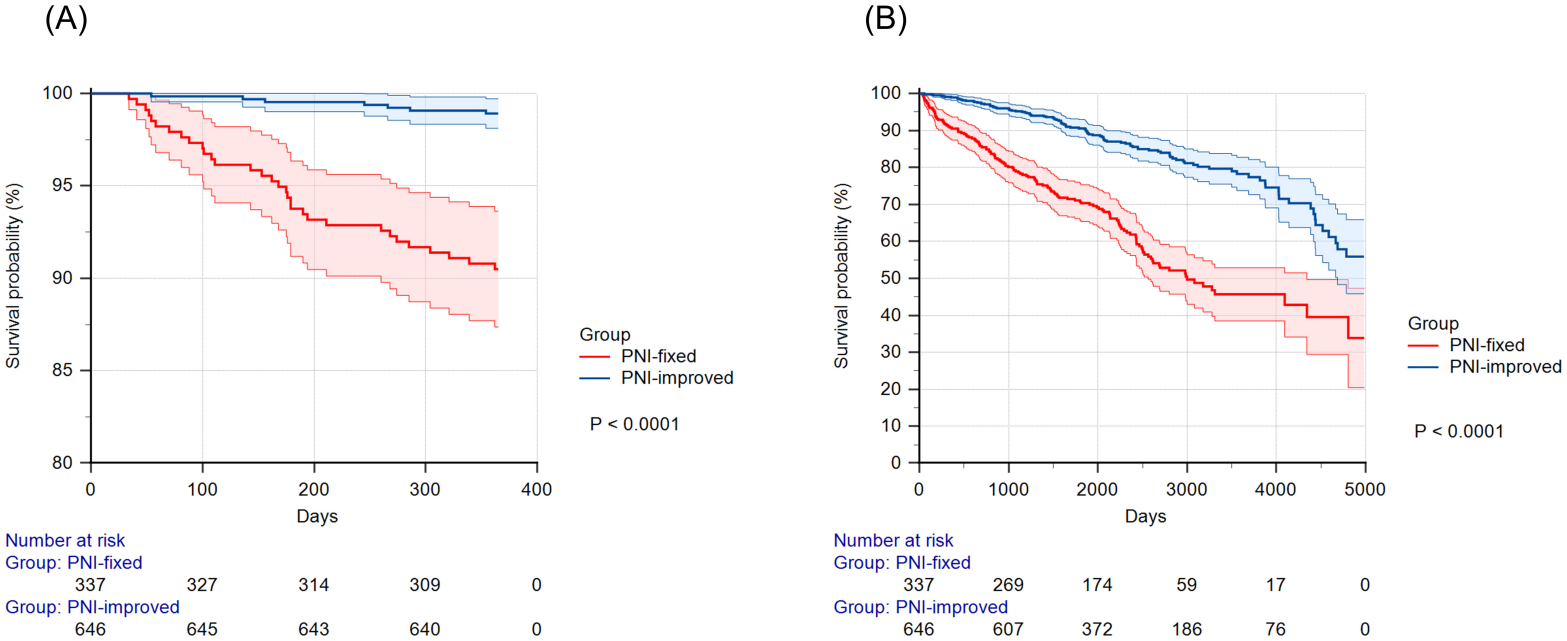
Kaplan–Meier survival curves according to PNI trajectory groups. (A) Kaplan–Meier curves for 1-year survival. (B) Kaplan–Meier curves for overall survival. The figures show the patients' Kaplan–Meier survival curves according to PNI trajectory groups. The red line represents the group with minimal changes in PNI values after surgery (PNI-fixed group). The blue line represents the group with a steep increase in PNI values after surgery (PNI-improved group). The shaded area represents the 95% confidence interval. PNI, prognostic nutritional index.

Figure 4 displays the Kaplan–Meier curves for 1-year and overall all-cause survival according to the PNI trajectory groups. The log-rank test showed that 1-year and overall survival probabilities were significantly lower in the PNI-fixed group compared to the PNI-improved group (*p* < 0.001).

## 4 Discussion

In the current study, we analyzed the changing patterns of postoperative PNI values after OPCAB surgery using a trajectory analysis method. Our findings indicated that patients who demonstrated significant improvements in PNI values early after surgery showed markedly lower 1-year and overall all-cause mortality rates than did those with minimal changes in PNI values. The PNI recovery pattern was identified as an independent predictor of 1-year and overall all-cause mortality, even after adjusting for confounding factors. Furthermore, incorporating the PNI trajectory patterns further enhanced the predictability of the regression model for 1-year mortality when compared to the model that included only individual preoperative and postoperative PNI values.

In patients undergoing cardiac surgery, postoperative malnutrition is a common complication (1–3). Underlying comorbidities, such as reduced cardiac function, can exacerbate intestinal dysfunction after surgery (1, 27), and inadequate nutritional support also contributes to the high prevalence of postoperative malnutrition after cardiac surgery (15). Therefore, screening for malnourished patients and providing nutritional support during the postoperative period is critical in this population. However, previous studies on nutritional monitoring have primarily focused on preoperative nutritional status, and there is no global consensus on postoperative nutritional monitoring and support, apart from the recommendation to initiate oral feeding early after surgery (18). Therefore, we examined the prognostic value of the postoperative recovery patterns of nutritional status. The Geriatric Nutritional Risk Index (GNRI), Controlling Nutritional Status (CONUT), and PNI are objective nutritional indices that have been widely studied in cardiac surgery. The GNRI incorporates albumin levels, ideal body weight, and actual body weight in its calculation. However, as postoperative body weight is greatly influenced by intraoperative fluid management (28, 29), the GNRI may not be an ideal tool for monitoring immediate postoperative nutritional status. The CONUT offers the advantage of incorporating cholesterol into its calculation alongside albumin and lymphocytes. Nevertheless, the widespread use of cholesterol-lowering medications in patients with coronary artery disease may significantly influence the trajectory pattern of CONUT scores after surgery. PNI was originally developed to assess the nutritional status of cancer patients, and its applicability in individuals with coronary artery disease has been subject to debate (30). However, a recent meta-analysis supports its utility as a prognostic marker in coronary artery disease patients (11). Moreover, PNI has the practical advantage of being easily calculated from routine blood test results, making it a convenient and suitable indicator for monitoring postoperative changes.

This study investigated the changing pattern of postoperative PNI values using trajectory analysis, a specialized statistical method for analyzing repeatedly measured data over time (20, 21). This approach uncovers meaningful patterns that static measures cannot capture and is being actively studied in various clinical fields as a useful tool to analyze the repeatedly measured biomarkers (22, 23), including in cardiovascular diseases (31, 32). In this study, our trajectory analysis of changing postoperative PNI patterns categorized patients into two distinct clusters: one group exhibited a steep increase in PNI values 1 month after surgery, while another showed minimal changes in PNI values compared to their values immediately after surgery. This distinction led to a stark contrast in 1-year and long-term overall all-cause mortality rates, with the “PNI-improved” group demonstrating markedly improved survival rates. The odds ratio of the PNI trajectory pattern for 1-year mortality was 7.931 (95% CI: 3.117–20.180), and the hazard ratio for overall mortality was 2.120 (95% CI: 1.579–2.845), which can be considered clinically meaningful results. Additionally, the PNI trajectory pattern remained an independent predictor of 1-year and overall mortality in multivariable models adjusted for confounding factors, including pre- and postoperative PNI values. Furthermore, the predictive power for mortality significantly improved when the PNI trajectory pattern was added to the multivariable regression model. These findings highlight the importance of the PNI recovery pattern, which cannot be captured by a single-point static PNI value, and provide novel insights into postoperative nutritional monitoring and support in OPCAB surgery. They also highlight the necessity of enhancing nutritional status in the early postoperative period after OPCAB surgery to improve prognosis.

As the PNI incorporates lymphocyte counts and albumin levels, postoperative PNI values can be significantly affected by the inflammatory reaction and intravascular volume changes. Therefore, we focused on patients undergoing OPCAB surgery to minimize the confounding influences of cardiopulmonary bypass regarding systemic inflammation and hemodilution (33, 34). Nevertheless, PNI values decreased after surgery in 92% (904/983) of patients compared to their preoperative values in this study, indicating the limited discriminative value of PNI values measured at a single point during the immediate postoperative period. Consequently, static postoperative PNI values measured at a single point were not an independent predictor of mortality in this study. Instead, observing PNI recovery patterns proved instrumental in identifying patients with a poor clinical course following OPCAB surgery.

Interestingly, the preoperative PNI value was significantly lower in the PNI-fixed group than in the PNI-improved group in the present study. This finding is consistent with a previous study reporting that patients with low preoperative PNI values had poorer nutritional status perioperatively after CABG surgery (13). In this context, it is assumed that inadequate immune function and nutritional supplementation before surgery may synergistically interact with postoperative suppression of the immune system and metabolic synthesis, potentially negatively affecting the patient's prognosis.

Our study has several strengths. First, the novel attempt to analyze the changing pattern of postoperative PNI using trajectory analysis may provide valuable insights into postoperative nutritional monitoring, which has previously been somewhat subjective, unclear, and often overlooked. Moreover, the significant impact of the PNI recovery pattern on mortality outcomes provides an important rationale for postoperative nutritional support, which may greatly influence survival. Additionally, a considerable number of patients were enrolled in the present study (*n* = 983), enhancing statistical reliability. Despite the above strengths, this study also has some limitations. First, due to the retrospective nature and single-center design of this study, the generalizability of the findings may be limited. Second, while our trajectory analysis offers a new methodological approach for evaluating nutritional changes, it inherently categorizes patients into broad groups, potentially overlooking subtle individual variations. Additionally, the results of the trajectory analysis may vary depending on the analysis tool owing to methodological differences (21). Nonetheless, trajectory analysis is based on rigorous statistical computations (20, 21), and has been widely validated in clinical studies as a reliable approach for characterizing heterogeneous temporal trends (22, 23, 31). While this method has inherent limitations, we believe that it provides a novel perspective on the dynamic patterns of postoperative nutritional recovery. Third, the incidence of 1-year mortality was relatively low, which may have restricted the statistical power for conducting multivariable regression analysis. Fourth, 636 patients with insufficient PNI data were excluded from the analysis, representing a significant proportion of the screened patients. However, the exclusion process was conducted without bias, and the analysis was performed on a robust sample of 983 patients after exclusions. Fifth, the analysis did not encompass other outcomes such as cardiac mortality or major adverse cardiac and cerebrovascular events (MACCE) due to data limitations. Future research should focus on investigating the impact of postoperative PNI changes on various outcomes, including cardiac mortality and MACCE. Finally, our study did not incorporate a comprehensive evaluation of postoperative nutritional status. Incorporating a wider range of nutritional parameters—such as caloric intake, body composition, and functional capacity—could provide a more in-depth understanding of nutritional recovery in future studies.

## 5 Conclusions

This study found that patients who demonstrated a meaningful improvement in their postoperative PNI values in the month following OPCAB surgery had significantly lower 1-year and overall all-cause mortality rates than patients with minimal changes in PNI values. Furthermore, the PNI recovery pattern was an independent predictor of 1-year and overall mortality, even after adjusting the confounding factors. Therefore, identifying the PNI recovery pattern following OPCAB surgery could help screen patients at high risk for mortality.

## Data Availability

The raw data supporting the conclusions of this article will be made available by the authors, without undue reservation.
