# Supplementary material for: Predictive value of postoperative prognostic nutritional index trajectory for mortality outcomes after off-pump coronary artery bypass surgery: a retrospective cohort study
Source: Front Nutr. 2025 May 7;12:1530651. doi: 10.3389/fnut.2025.1530651 (PMC12092210; doi:10.3389/fnut.2025.1530651)
Supplement: Supplementary file 1 [file Table_1.docx]

***Supplementary Tables***

**Supplementary Table S1.** Number of patients and the average probability of assignment according to the number of clusters in trajectory analysis

| Number of Clusters | Number of patients | | | | | Average probability of assignment (%) | | | | |
| --- | --- | --- | --- | --- | --- | --- | --- | --- | --- | --- |
|  | Cluster1 | Cluster2 | Cluster3 | Cluster4 | Cluster5 | Cluster1 | Cluster2 | Cluster3 | Cluster4 | Cluster5 |
| 2 | 646 | 337 |  |  |  | 94.43 | 91.83 |  |  |  |
| 3 | 523 | 232 | 228 |  |  | 87.81 | 85.5 | 92.34 |  |  |
| 4 | 346 | 312 | 182 | 143 |  | 79.38 | 81.7 | 83.85 | 91.57 |  |
| 5 | 286 | 228 | 202 | 140 | 127 | 78.27 | 81.84 | 76.85 | 91.39 | 86.98 |

**Supplementary Table S2.** Univariable logistic regression analysis of chosen variables for predicting one-year mortality.

| Variable | Odds ratio  (95% CI) | p-value |
| --- | --- | --- |
| PNI trajectory pattern |  |  |
| Improved | Reference |  |
| Fixed | 9.578 (4.180, 21.945) | <0.001 |
| Pre-PNI | 0.911 (0.871, 0.952) | <0.001 |
| Post-PNI | 0.912 (0.828, 1.005) | 0.063 |
| EuroSCORE II | 1.132 (1.000, 1.280) | 0.049 |
| Sex (Female) | 1.213 (0.594, 2.474) | 0.596 |
| Age (years) | 1.093 (1.045, 1.143) | <0.001 |
| Body mass index (kg/m^2^) | 0.841 (0.757, 0.935) | 0.001 |
| Emergency | 3.663 (1.044, 12.844) | 0.043 |
| Hypertension | 0.746 (0.372, 1.496) | 0.409 |
| Chronic renal failure | 1.841 (0.899, 3.772) | 0.095 |
| CVA history | 0.778 (0.300, 2.023) | 0.607 |
| Diabetes mellitus | 0.778 (0.410, 1.477) | 0.443 |
| Congestive heart failure | 1.237 (0.508, 3.013) | 0.640 |
| COPD | 3.324 (1.234, 8.951) | 0.018 |
| Recent MI | 1.417 (0.732, 2.740) | 0.301 |
| Anemia | 5.690 (2.362, 13.706) | <0.001 |
| WBC (/μl) | 1.000 (1.000, 1.000) | 0.791 |
| Platelet (x10^3^/μl) | 0.996 (0.991, 1.001) | 0.116 |
| Glucose (mg/dL) | 0.994 (0.987, 1.001) | 0.086 |
| CK-MB (μg/L) | 0.999 (0.988, 1.010) | 0.895 |
| Ejection fraction (%) | 0.969 (0.949, 0.990) | 0.004 |
| Graft number | 0.914 (0.636, 1.313) | 0.626 |
| Operation time (min) | 1.002 (0.996, 1.009) | 0.479 |
| Fluid Input/Output (ml) * | 1.000 (1.000, 1.000) | 0.882 |
| Bleeding (ml) | 1.000 (0.999, 1.001) | 0.794 |
| Perioperative Transfusion | 3.088 (1.450, 6.576) | 0.003 |

* Fluid Input/Output was defined as intraoperative fluid input minus urine output. Abbreviations: PNI, prognostic nutritional index; CVA, cerebrovascular accident; COPD, chronic obstructive pulmonary disease; MI, myocardial infarction; WBC, white blood cell; RAS inhibitor, renin-angiotensin system inhibitor; CK-MB, Creatine Kinase-MB

**Supplementary Table S3.** Univariable Cox regression analysis of chosen variables for predicting overall mortality.

| Variable | Hazard ratio  (95% CI) | p-value |
| --- | --- | --- |
| PNI trajectory pattern |  |  |
| Improved | Reference |  |
| Fixed | 3.148 (2.450, 4.046) | <0.001 |
| Pre-PNI | 0.925 (0.909, 0.941) | <0.001 |
| Post-PNI | 0.916 (0.881, 0.952) | <0.001 |
| EuroSCORE II | 1.174 (1.125, 1.225) | <0.001 |
| Sex (Female) | 1.032 (0.779, 1.367) | 0.827 |
| Age (years) | 1.059 (1.042, 1.076) | <0.001 |
| Body mass index (kg/m^2^) | 0.926 (0.890, 0.963) | <0.001 |
| Emergency | 1.486 (0.734, 3.010) | 0.271 |
| Hypertension | 1.328 (0.977, 1.807) | 0.070 |
| Chronic renal failure | 2.126 (1.601, 2.824) | <0.001 |
| CVA history | 1.622 (1.197, 2.199) | 0.002 |
| Diabetes mellitus | 1.495 (1.156, 1.932) | 0.002 |
| Congestive heart failure | 1.722 (1.256, 2.360) | 0.001 |
| COPD | 3.433 (2.265, 5.202) | <0.001 |
| Recent MI | 1.218 (0.945, 1.571) | 0.128 |
| Anemia | 2.677 (2.052, 3.494) | <0.001 |
| WBC (/μl) | 1.000 (1.000, 1.000) | 0.685 |
| Platelet (x10^3^/μl) | 1.000 (0.998, 1.002) | 0.916 |
| Glucose (mg/dL) | 1.001 (0.999, 1.003) | 0.273 |
| CK-MB (μg/L) | 0.994 (0.977, 1.010) | 0.450 |
| Ejection fraction (%) | 0.975 (0.967, 0.983) | <0.001 |
| Graft number | 0.864 (0.747, 0.998) | 0.047 |
| Operation time (min) | 0.999 (0.996, 1.002) | 0.485 |
| Fluid Input/Output (ml) * | 1.000 (1.000, 1.000) | 0.480 |
| Bleeding (ml) | 1.000 (0.999, 1.000) | 0.352 |
| Perioperative Transfusion | 1.904 (1.470, 2.466) | <0.001 |

* Fluid Input/Output was defined as intraoperative fluid input minus urine output. Abbreviations: PNI, prognostic nutritional index; CVA, cerebrovascular accident; COPD, chronic obstructive pulmonary disease; MI, myocardial infarction; WBC, white blood cell; RAS inhibitor, renin-angiotensin system inhibitor; CK-MB, Creatine Kinase-MB
